# Subcutaneous Emphysema Following Tonsillectomy: A Rare Complication of a Common Surgery

**DOI:** 10.7759/cureus.53825

**Published:** 2024-02-08

**Authors:** Abdulrahman F Alzamil, Fahad K Bedawi, Danya A AlAseeri, Ebrahim Almulla, Hesham Y Alrayyes, Andrew Riskalla

**Affiliations:** 1 Otolaryngology - Head and Neck Surgery, Bahrain Royal Medical Services, Riffa, BHR; 2 Otolaryngology - Head and Neck Surgery, King Hamad University Hospital, Manama, BHR

**Keywords:** iatrogenic subcutaneous emphysema, surgical emphysema, post-tonsillectomy complication, emphysema post tonsillectomy, subcutaneous emphysema

## Abstract

Post-tonsillectomy emphysema is an infrequent yet critical complication that follows tonsillectomy - a prevalent surgical procedure for treating conditions like recurrent tonsillitis and obstructive sleep apnea. While tonsillectomy is generally safe, it is not without risks, including the rare occurrence of postoperative emphysema, where air accumulates abnormally in the neck and head's soft tissues, potentially leading to severe respiratory distress. We present a case of a middle-aged female who underwent tonsillectomy and subsequently developed symptoms indicative of post-tonsillectomy emphysema. Diagnosed through a combination of physical examination and imaging, her treatment involved conservative management and careful monitoring, ultimately resulting in full recovery without the need for surgical intervention.

## Introduction

Tonsillectomy, the surgical removal of the tonsils, is one of the most commonly performed otolaryngological procedures worldwide, primarily indicated for the treatment of recurrent tonsillitis, obstructive sleep apnea, and other related conditions [1]. While tonsillectomy is generally considered safe, various postoperative complications may arise, ranging from minor complications such as postoperative pain and minor bleeding to more severe and rare complications [2]. One such rare complication that has been documented in the medical literature is post-tonsillectomy emphysema, a condition characterized by the presence of air in the neck and soft tissues of the head and neck region [3].

Post-tonsillectomy emphysema is a noteworthy complication due to its potential to cause respiratory distress with subsequent life-threatening consequences. It typically occurs within the immediate postoperative period or shortly thereafter and is thought to result from the forced entry of air into the cervical soft tissues during the procedure or subsequently secondary to forceful straining [4]. The accumulation of air in these tissues can lead to significant discomfort, pain, and swelling of the neck, potentially impeding the patient's airway, and necessitating immediate intervention [5,6].

This case report aims to present a clinical case of post-tonsillectomy emphysema, including its clinical presentation, diagnostic evaluation, management, and outcomes. The documentation of such cases is essential for a better understanding of the pathophysiology and management of post-tonsillectomy emphysema, ultimately contributing to improved patient safety and surgical outcomes.

## Case presentation

A 45-year-old female presented to the otolaryngology clinic with recurrent tonsillitis requiring multiple use of antibiotics. The patient’s weight was 66 kilograms and known type 2 diabetes mellitus on oral anti-glycemic medication, otherwise no medical issues. On examination, she had grade 3 cryptic tonsils and thus was booked for surgery. She underwent surgery under general anesthesia with an endotracheal tube. Intubation was smooth, uneventful, and atraumatic. Tonsillectomy using bipolar cautery technique was done. Tonsils were found to be fibrosed and adherent imposing difficulty in dissection. Recovery from anesthesia was unremarkable, and the patient was extubated smoothly.

**Figure 1:**
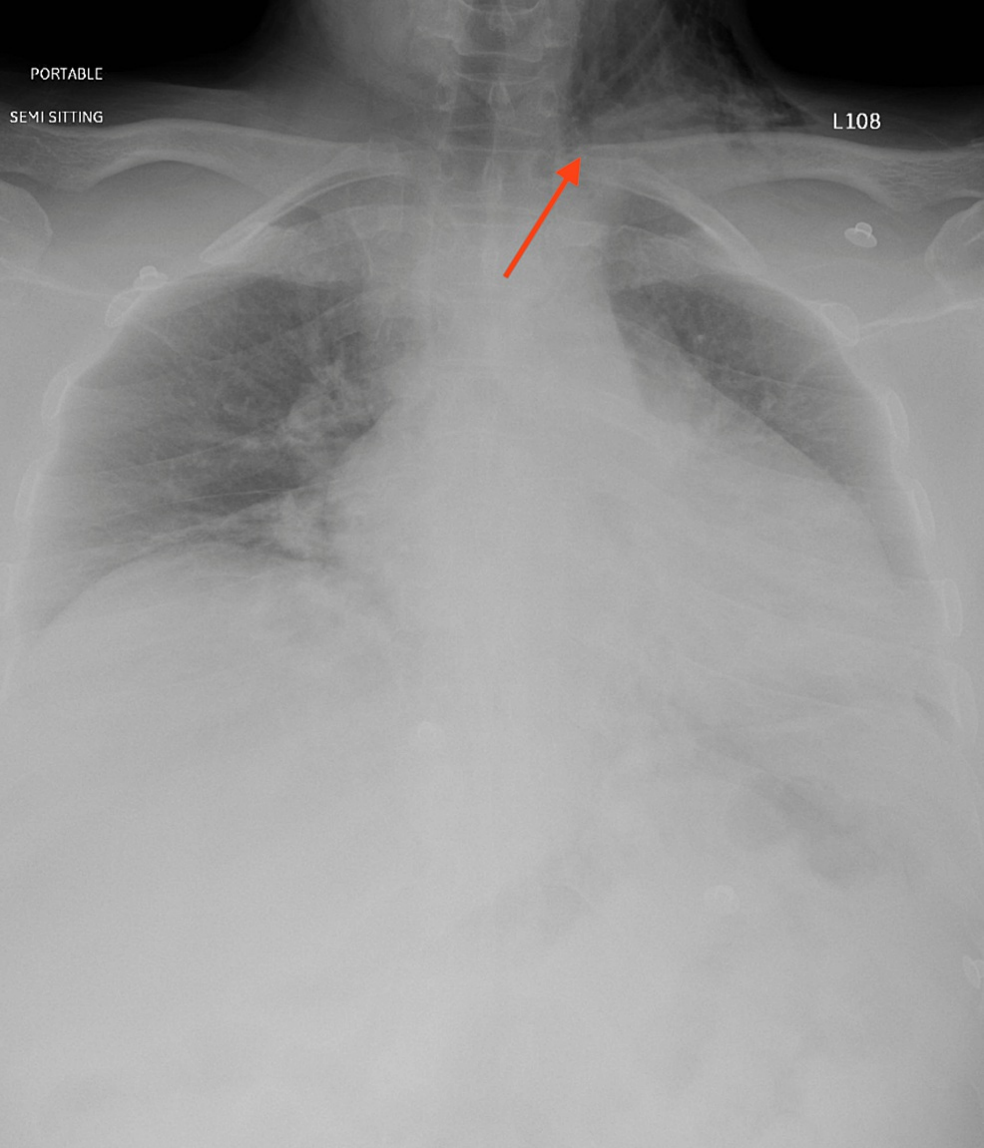
The plain anterior-posterior (AP) X-ray The arrow pointing to soft tissue lucency on the left side of the neck.

**Figure 2:**
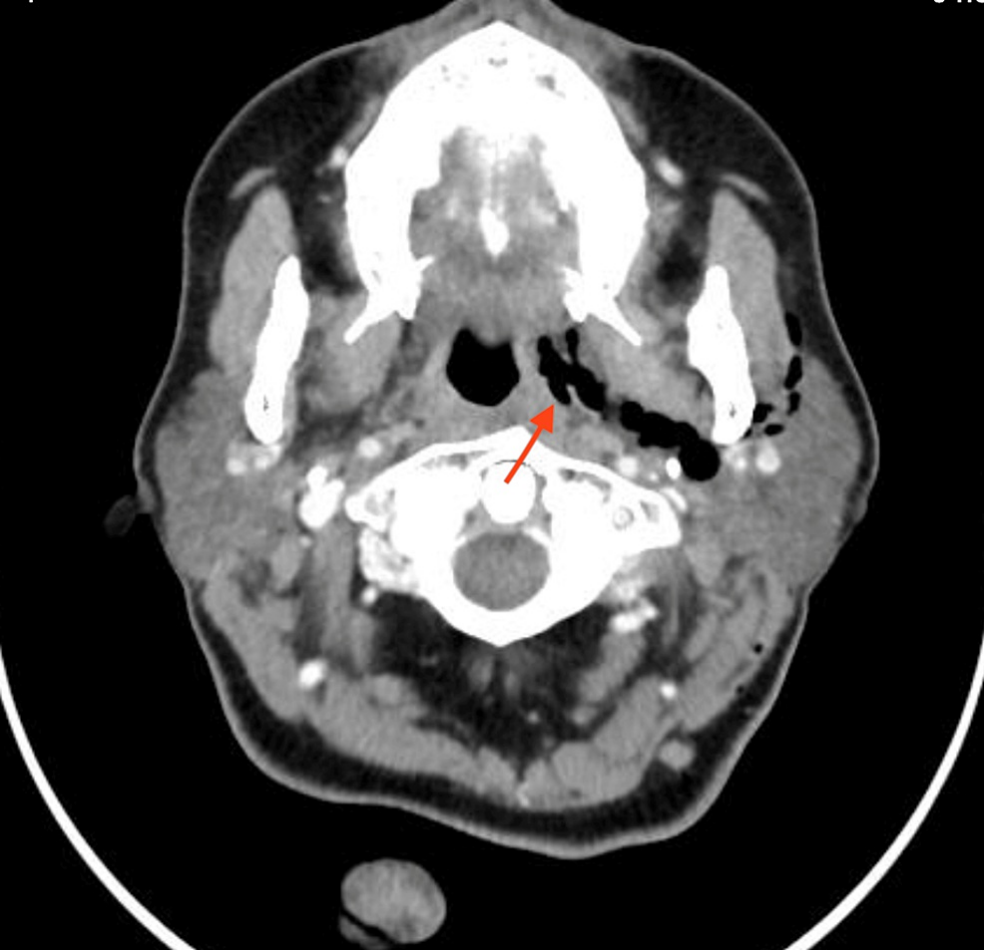
Computed tomography (CT) with contrast, axial view The arrow pointing to the area of extension of air to the left parapharyngeal space.

**Figure 3:**
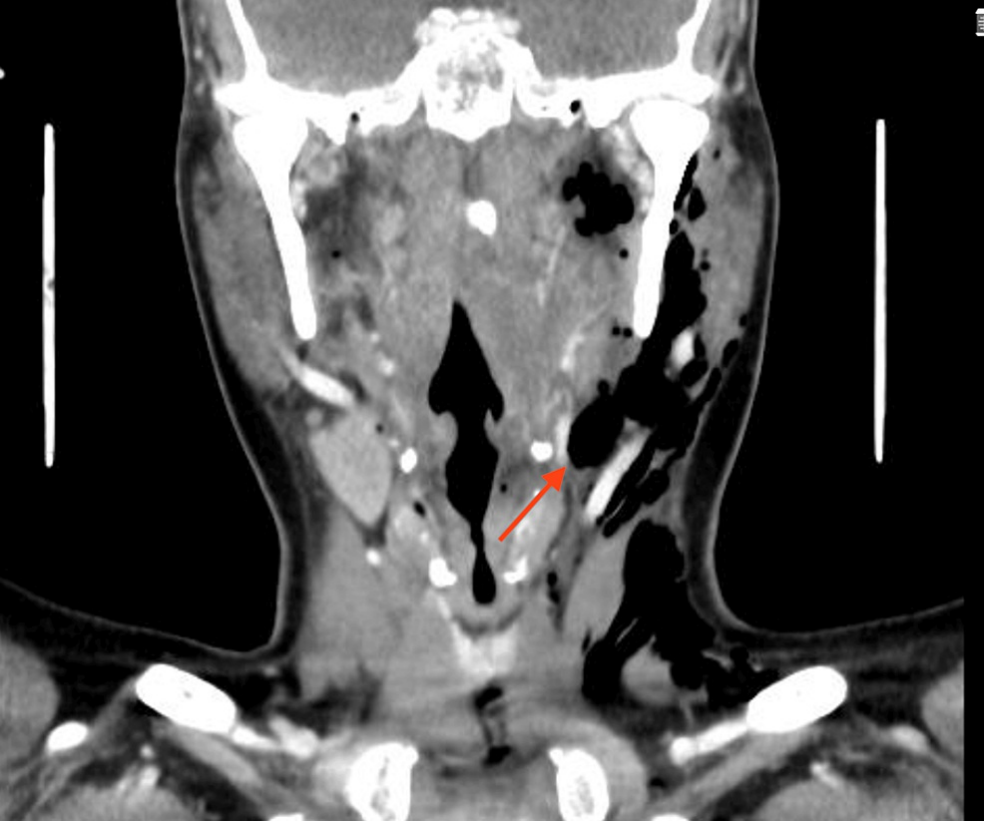
CT with contrast, coronal view The arrow pointing to air extending to parapharyngeal space into the rest of the neck spaces on the left side.

She presented to the emergency the next day with complaints of shortness of breath and neck pain radiating to her ears. She was vitally stable, with a saturation of 100% on room air, and had no history of fever, vomiting, or bleeding. After the oral examination, a healthy-looking slough was seen, with no point of bleeding. Crepitus was appreciated when palpating over the left side of the upper neck and submandibular region. A chest X-ray was done which showed air trapped in the left lateral neck (Figure 1). The patient was admitted and was kept NPO (Nil Per Os), intravenous broad-spectrum antibiotics were initiated, and a nasogastric tube was inserted for nutrition with a close vital monitor for any vascular or respiratory compromise. CT neck was done to confirm the presence of tracking from the tonsillar bed. It showed air pockets extending from the left tonsillar bed to parapharyngeal space, the subcutaneous and facial planes of the neck and chest wall, and the superior pneumomediastinum (Figures 2, 3). During her admission, she was clinically stable and found to improve daily with reduced crepitus progressively on a daily basis. She was kept under surveillance for cardio-thoracic surgery as well, which established that no surgical intervention was indicated. X-ray was repeated on day 6 of admission and showed complete resolution or regression of emphysema and discharged in stable condition. She presented to the clinic after one week of discharge for follow-up and was doing well with no complaints with normal healthy slough on bilateral tonsillar beds.

## Discussion

Subcutaneous emphysema is an infrequent occurrence following tonsillectomy and has been documented in medical literature since as early as 1933 [5]. The exact process leading to subcutaneous emphysema and pneumomediastinum following tonsillectomy is not well-documented. Some reports suggest that injury to the pharyngolaryngeal mucosa, either during surgery or intubation, can be responsible for this condition [4].

Tonsillectomy is considered one of the most common and safest otorhinolaryngology procedures, but they carry their own complications as any other procedures. A rare complication is cervical subcutaneous emphysema [5]. It is suspected that loss of mucosal integrity because of excessive dissection due to fibrosis and embedded tonsils, aggravated by an increase in intra-pharyngeal pressure, for example, due to positive pressure ventilation of anesthesia or coughing and vomiting by the patient can lead to such complications. Air leaks through the superior constrictor muscle, extending into the parapharyngeal and retropharyngeal spaces. If an excessive amount of air enters such planes, due to its communications and connections, can reach the mediastinum and even the peritoneum cavity causing cardio-respiratory compromise [6-8]. Fortunately, our patient had it only to the neck and upper mediastinum without cardiological or respiratory compromise. Initial diagnosis is very important, as the patient can present from mild neck discomfort with crepitation, to pneumothorax and pneumomediastinum with cardiorespiratory issues. Imaging is very important for the diagnosis. Plain chest and neck X-rays are very helpful. In a review of the literature, CT scans are recommended as the best confirmatory diagnosis modality.

The management of subcutaneous emphysema is typically conservative, as it tends to resolve on its own in most cases. Supplemental oxygen can help facilitate the absorption of nitrogen present in the trapped air, creating a favorable concentration gradient for the gas to move downward.

Patients should receive intravenous hydration and pain relief, especially if they experience odynophagia. It is advisable for patients to avoid actions that increase pressure in the affected area, such as coughing, vomiting, and straining, until the emphysema resolves. When necessary, medications like cough suppressants, antiemetics, and stool softeners can be prescribed. To prevent potential contamination and infection from the oral cavity, broad-spectrum antibiotics should be administered. This precaution helps maintain the overall well-being of the patient during the healing process. Furthermore, keeping the patient NPO is imperative to reduce the chance of air swallowing and increasing upper airway pressure [9-11]. In a few cases, cardiorespiratory compromise ensued requiring tracheostomy and inserting chest tubes to relieve pressure and maintain circulation of such systems [6,9,12].

## Conclusions

In conclusion, emphysema is a very rare complication after tonsillectomy that is thought to occur due to loss of integrity of mucosa which can be aggravated by increased pressure from the anesthesia side or due to patient factors from coughing and vomiting. Prompt diagnosis and careful and frequent reassessment are mandatory, but such conditions usually resolve by themselves.
